# Management Implications of the Biodiversity and Socio-Economic Impacts of Shrimp Trawler By-Catch in Bahía de Kino, Sonora, México

**DOI:** 10.1371/journal.pone.0035609

**Published:** 2012-06-18

**Authors:** Lorayne Meltzer, Naomi S. Blinick, Abram B. Fleishman

**Affiliations:** 1 Environmental Studies Faculty, Prescott College, Prescott, Arizona, United States of America; 2 Research and Conservation Program, Prescott College Kino Bay Center for Cultural and Ecological Studies, Bahía de Kino, Sonora, México; Institute of Marine Research, Norway

## Abstract

The shrimp fishery is the most economically important fishery in Mexico. The trawler-based portion of this fishery results in high rates of by-catch. This study quantifies and describes the biodiversity of by-catch associated with trawling in the Bahía de Kino region of Sonora, Mexico. Data were collected from 55 trawls, on six boats, over 14 nights, during November of 2003, 2004, 2006–2009. By-catch rates within trawl samples averaged 85.9% measured by weight. A total of 183 by-catch species were identified during the course of this study, including 97 species of bony fish from 43 families, 19 species of elasmobranchs from 12 families, 66 species of invertebrates from eight phyla, and one species of marine turtle; seven of the documented by-catch species are listed on the IUCN Red List, CITES, or the Mexican NOM-059-ECOL-2010; 35 species documented in the by-catch are also targeted by local artisanal fishers. Some of the species frequently captured as juveniles in the by-catch are economically important to small-scale fishers in the region, and are particularly sensitive to overexploitation due to their life histories. This study highlights the need for further research quantifying the impacts of high levels of by-catch upon small-scale fishing economies in the region and presents strong ecological and economic rationale for by-catch management within the shrimp fishery of the Gulf of California. Site-specific by-catch management plans should be piloted in the Bahía de Kino region to address the growing momentum in national and international fisheries policy regimes toward the reduction of by-catch in shrimp fisheries.

## Introduction

The United Nations Food and Agriculture Organization (FAO) reports that the shrimp fishery is the most important fishery in Mexico in terms of value, exports, and employment [1]. According to world averages compiled by the FAO, global shrimp trawling results in an estimated 62.3% by-catch, the highest by-catch rate of any fishing industry [2] and produces at least one third of global fisheries discards [2]–[4]. This paper addresses the shrimp trawl fishery in Sonora; previous studies have documented by-catch rates between 70 and 97% in this region [5]–[7].

**Figure 1:**
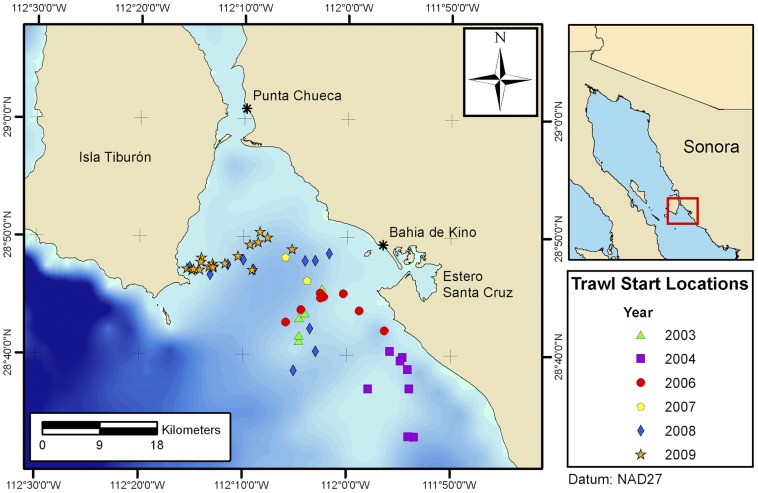
Locations of 53 sampled trawls in the Bahía de Kino region, Sonora, Mexico, 2003–2009. Start locations of sampled trawls between 2003 and 2009. The sampled trawls occurred between 15.2 m and 39 m, typically along a sandy substrate.

In 2009, the Mexican shrimp fishery landed 196,456 metric tons (MT) of shrimp (all weights listed are live weight of catch). Sonora produces more shrimp overall (aquaculture and trawling combined) than any other state in Mexico, producing 101,045 MT of shrimp in 2009, which represented 51.4% of the national shrimp yield for that year (Fig. 1). In response to increasing market demand, Sonora’s shrimp production has increased nearly 400% since 1999, when 25,538 MT of shrimp were landed [8]. This dramatic increase is due to explosive development in shrimp production through aquaculture.

The Mexican shrimp fishery is comprised of small boats operating in bays and estuaries, otter trawlers operating in the open sea, most commonly in waters from 11–45 meters deep, and aquaculture [1]. The state of Sonora leads the production of cultivated shrimp in Mexico, and is second only to its southerly neighbor state Sinaloa in the landings of trawled shrimp. In 2009, 63.6% (84,798 MT) of all Mexican cultivated shrimp were produced in Sonora, and 26.0% (10,970 MT) of Mexican trawled shrimp were landed in Sonora. Although the majority of shrimp produced in Mexico now comes from aquaculture (67.8% of national shrimp production in 2009), trawling still represents a sizable economic force and has significant environmental and socioeconomic impacts [8].

The otter trawlers that characterize the modern industrialized offshore shrimp fishery range from 18 to 25 m in length, are powered by 220 to 620 hp diesel engines, can remain at sea for more than 15 days, trawl from 5.5 to 110 meters, and can hold as much as 100 MT of shrimp frozen in on-board refrigeration systems. They are also equipped with navigational instruments: GPS, compass, and sonar. The funnel-like net is held open by two large wooden boards. A chain runs between these boards and is dragged across the sea floor to disturb the shrimp into the net. The trawlers are equipped with twin nets with a headline of 23–36 m and a mesh size of 3.81–4.31 cm in the cod end [1], [9], [10]. In 2009 the Sonoran shrimp trawling fleet consisted of 484 boats, 374 of which were 20–25 meters in length, 87.6% of which had a product capacity of more than 40 tons, and ∼92% of which are more than 20 years old [8]. The fleet characteristics, combined with documented declines in catch per unit effort (CPUE) define this fleet as inefficient and overcapitalized [9], [11]. In 2005, in recognition of the over-capitalized fleet, the federal government began the *Programa de Retiro Voluntario de Embarcaciones Camaroneras* (Voluntary Retirement Program for Shrimp Vessels), which has been largely successful. Between 2005 and 2011, the program has spent approximately 46 million USD to retire 527 vessels, 38.5% of the national fleet in Mexico [12].

Turtle Exclusion Devices (TEDs) have been required on the Pacific coast of Mexico since 1996 [1]. Mexico is among the 14 countries that currently meet the U.S. Department of State standards for the use of TEDs [13]. Meeting this standard is necessary for Mexico to maintain the economically crucial ability to export shrimp to the United States. However, on all but one boat visited during this study TEDs were removed and not used during fishing efforts. U.S. Public Law 101–162, Section 609, prohibits the importation of shrimp harvested in a way that may adversely affect sea turtles; nations that have been certified to harvest shrimp with comparable programs to the U.S. to protect sea turtles (such as using TEDs) are exempt from the ban. After finding that Mexico’s TED program no longer met the standards of Section 609, the United States Department of State withdrew Mexico’s certification to import trawled shrimp into the U.S. on March 24^th^, 2010. The ban went into effect on April 20, 2010 [13]. After intensive training efforts for trawler crews, captains, and federal enforcement agents during the summer of 2010, Mexico was recertified by the U.S. Department of State under Section 609 on October 15^th^, 2010. The use of TEDs subsequently increased.

Though not extensively quantified in the Gulf of California, significant direct and indirect ecological impacts have been documented as a result of shrimp fisheries in Mexico and around the world. Impacts specifically from trawler by-catch include: high mortality of captured individuals, including juvenile individuals and threatened and sensitive species; changes in species composition and benthic community structure; and changes in organic nutrient loading and oxygen levels due to decomposition of discards [1], [14], [15], [16]. The additional impacts of trawling to benthic habitats include alteration of physical structure and sediment suspension [1], [17], [18].

Efforts to document the effects of shrimp trawling on habitats and community composition have increased the general knowledge in both the Gulf of California and globally. Three previous studies quantifying by-catch in the Sonora/Sinaloa region documented 241 species of fish from 65 families with a fish by-catch rate of 70% [7], 105 species of fish from 52 families and a by-catch rate of 90.6% [6] and 87 species of fish from 43 families representing a 82.8% by-catch rate [5]. A study of by-catch from experimental trawls in the southeastern Gulf found 209 species of fish and a fish by-catch rate of 83–97% [19], while a study of experimental trawl catch off the mouth of Rio Baluarte in the state of Sinaloa documented 143 species from five phyla [20]. Another study conducted throughout the Gulf documented 243 by-catch species from experimental trawls in 2002, 2005, and 2007 [21]. Our study is the only one to document the diversity of trawler by-catch for all associated faunal groups over several years specifically along the central Sonoran coast during the open shrimp season. Our study strengthens the conclusions from the abovementioned studies, and presents a strong case for by-catch management.

In addition to its ecological impacts, the high incidence of commercial species in the by-catch has led to negative socio-economic impacts on small-scale fisheries worldwide [1], [22]. Shrimp trawling fleets negatively impact small-scale fisheries and local communities and economies through direct competition for the same resources, habitat disturbance, and the by-catch and discard of juveniles of important commercial species. This conflict between commercial shrimp trawlers and small-scale fishers has been documented previously on both global and regional scales [1], [16], [22]. As shrimp trawlers in Mexico are not licensed to sell anything other than shrimp, all other captured species are by-catch and are unregistered, unregulated, unmonitored and mostly unused, although individuals of commercial species that are of marketable size will be retained for personal consumption or to be sold illegally. The principle goal of this study was to provide data and analysis of species diversity of the by-catch associated with shrimp trawling in the Bahía de Kino region in order to inform management and local small-scale fishers of the ecological and socio-economic impacts.

In order to suggest specific management actions, the objectives of the study were: to develop a cumulative list and catalog of by-catch species documented over the duration of the study; to record the diversity of species listed as having conservation status under Mexican NOM-059-ECOL-2010, listed on the International Union for the Conservation of Nature (IUCN) Red List, or protected under the Convention on the International Trade of Endangered Species of Wild Flora and Fauna (CITES); to identify species common in the by-catch whose life histories are either highly unknown, or point to vulnerability from this type of extraction; to describe the incidence of commercial species in the by-catch; and to provide spatial and temporal data to determine if there are areas, depths, or times of night that result in significantly different rates of by-catch.

## Methods

### Ethics Statement

Monitoring of by-catch species associated with commercial shrimp trawling was conducted in accordance with relevant national laws as specified in the Mexican Ley de Pesca (National Fisheries Law) and monitoring protocols reviewed in the literature and developed in collaboration with regional fisheries biologists. We were on board the shrimp trawlers with the permission of the Port Captain and with the support of the captains of the individual vessels on which we worked.

### Study Area

Bahía de Kino is a shallow bay located on the coast of Sonora, in the Gulf of California. Shrimp trawlers most commonly access this region from the Port of Guaymas. The bottom consists of alluvial deposits from the broad coastal plains that line the adjacent coast [23]. The bay is also influenced by sedimentation from Estero Santa Cruz, a 3,622 hectare mangrove lagoon southeast of the town of Bahía de Kino, which was historically the terminal of the Rio Sonora [24]. The inshore environment in this region is composed of a shallow, sloping bottom reaching a maximum depth of approximately 80 m between the coast of Bahía de Kino and Isla Tiburón, as well as off the southern tip of Isla Dátil [23]. Nutrient production in the region is generally high, due to year-round upwelling around the Midriff Islands [25].

For this study, the Bahía de Kino region was defined as 28°50′00″N to 28°30′00″N, and between 112°16′00″W and 111°50′00″W (Fig. 1). The marine area where the sampled trawls took place is bordered to the north by the Canal de Infiernillo, to the east by the coastlines of Bahía de Kino and Punta San Nicholas, and to the west by Islas Tiburón and Dátil. The sampled trawls were between depths of 15.2 m and 39 m, typically along a sandy substrate.

### Methods

Observer teams of one researcher and 2–4 undergraduate students participated in two days of onshore training in species identification and onboard sampling protocol. Each team spent one night aboard a shrimp trawler working in the Bahía de Kino vicinity, for a total of 2–4 nights of sampling in November of 2003, 2004, 2006, 2007, 2008, and 2009.

General information recorded about each trawl set included; trawl number; trawl duration; trawl location (start and stop GPS locations); processed (head-off) shrimp weight for the trawl; by-catch species, and numbers retained by the crew; weight of sample per trawl; and weight (head – on) of shrimp in sample. To determine the rate of by-catch, a sample of 10 large shovelfuls was taken from the entire catch of each trawl as soon as it was released from the codends onto the deck. The weight of shrimp and the weight of the by-catch were then measured and the by-catch rate was calculated using the formula: weight of shrimp in sample/weight of all other organisms in sample. For the purposes of this study, “by-catch” was defined as all non-target animals.

By-catch diversity over the course of the study was gathered by recording every species other than target shrimp (*Farfantepenaeus californiensis, Litopenaeus stylirostris, L. vannamei*) present in the catch. A photograph was taken, right side down on a measured fish board, of every species identified. This information was compiled to develop a record of every by-catch species documented throughout the duration of the study, and which year(s) they were documented in the catch. In order to determine the impact of trawler by-catch on small-scale fisheries in the region, by-catch diversity data were cross-referenced with published accounts of species targeted by local small-scale commercial fishermen. ANOVA analysis was done to determine differences in mean by-catch rates by set number; one-tailed and two-tailed correlation tests were used to determine difference in mean by-catch rates by set depth.

## Results

Data were collected from 55 trawls, on six boats, over 14 nights, during November of 2003, 2004, 2006–2009. A total of 183 by-catch species were identified, including 97 species of bony fish from 43 families, 19 species of elasmobranchs from 12 families, 66 species of invertebrates from 8 phyla, and one species of marine turtle. Seven species protected under NOM-059-ECOL-2010, CITES, or listed on the IUCN Red List were identified in the by-catch [26]–[28] (Table 1). Out of the 183 total by-catch species, 35 have commercial value to local artisanal fishers [29]. By-catch data from all years were compiled in a master list (Table S1). Over the course of the study, photographs were taken of every species and compiled into a catalog to be used for training and onboard reference by observer teams. In 2009, taxonomy experts reviewed the cumulative photo catalog to ensure correct species identification (pers comm. Lloyd Findley and Richard Brusca 2009).

**Table 1:** By-catch species listed under Mexican endangered species legislation, the IUCN Red List, and/or CITES.

Latin Name	Common Name	NOM-059-ECOL-2010	IUCN Red List	CITES
*Totoaba macdonaldi*	Totoaba	P (endangered), endemic	Critically Endangered	Appendix I
*Caretta caretta*	Loggerhead Turtle	P (endangered), non-endemic	Endangered	Appendix I
*Hippocampus ingens*	Pacific Seahorse	Pr (Protected), non-endemic	Vulnerable	Appendix II
*Rhinobatos productus*	Shovelnose Guitarfish	Not listed	Near Threatened	Not listed
*Rhinoptera steindachneri*	Golden Cownose Ray	Not listed	Near Threatened	Not listed
*Sphyrna lewini*	Scalloped Hammerhead	Not listed	Endangered	Not listed
*Squatina californica*	Pacific Angel Shark	Not listed	Near Threatened	Not listed

**Figure 2:**
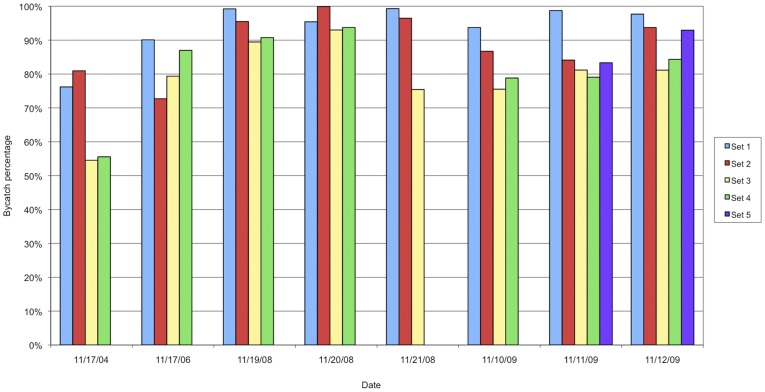
Rate of by-catch by set, from 33 samples from eight nights in the Bahía de Kino region, during 2004–2009. There is a significant difference in the average by-catch rate when the time of the trawls are compared (F = 6.9, p<.01). The first trawl of each night (light blue bars) sampled had the highest rate of by-catch, while set 3 (yellow bars) had the lowest rate. The third trawl of the night began on average between 3am and 4am.

Shrimp and by-catch weight was measured for samples taken from 33 trawls over the course of the study. By-catch rates, calculated from those 33 trawls, ranged from 54%–99.24% by weight, the average by-catch rate was 85.9% (Fig. 2).

One-tailed correlation tests show a barely significant negative correlation of r = −.28, p = .079 between depth and target shrimp weight, but there is no correlation between depth and by-catch rate. There are no significant correlations between these variables using two-tailed tests. Previous studies also found no correlation between depth and shrimp landings [5],[6]. ANOVA analysis shows a significant difference in the average by-catch rate when the time of the trawls are compared (F = 6.9, p<.01). The first trawl of each night sampled had the highest rate of by-catch, while the third set had the lowest. The third trawl of the night began on average between 3am and 4am. There was no significant difference in shrimp catch versus the time of trawl.

## Discussion

Shrimp trawling in the Bahía de Kino region of the Gulf of California results in remarkably high rates of by-catch. Applying our by-catch rate of 85.9%, it is estimated that 67,008 MT of by-catch resulted from the Sonoran trawled shrimp catch of 10,970 MT reported by SAGARPA in 2009 [8]. By-catch mortality of commercially valuable species would seemingly lead to direct negative impacts on the small-scale fisheries and local economies of Bahía de Kino, exacerbated by the high incidence of juveniles observed (but not quantified) in our study. Previous studies also document the high incidence of juvenile fish in the by-catch both regionally and globally [1], [5], [6], [16]. Our results show the urgent need for future studies to quantify the abundance of commercially important species in the by-catch, the abundance and diversity of juveniles in the by-catch, and the specific economic impacts of by-catch mortality on small-scale fisheries in the region. Further research is also indicated to reveal the cause of the reduced by-catch rates found during the third trawl of the night (1–4am).

Some of the species frequently encountered as juveniles in the by-catch are both highly valued by commercial small-scale fishers in the region and also particularly sensitive to overexploitation because of their life histories (i.e. long-lived species are more vulnerable because they are more likely to be killed as by-catch before reproducing). While more life-history information is needed to determine the effect of this by-catch mortality for most of the by-catch species; the Shovelnose Guitarfish, (*Rhinobatos productus*) is a fairly well-studied species in the Gulf of California and presents a convincing case for the need for by-catch management within the trawl shrimp fishery in Sonora.

*R. productus* is listed as threatened on the IUCN Red List [30] and has high commercial value to fishers in Bahía de Kino. It is often taken as by-catch by the Gulf shrimp-trawl fishery [31] and has been observed in the by-catch in every year of this study. Our continuing studies show that the majority of landed individuals being below the minimum known reproductive size in the region (unpubl. data). As with most elasmobranchs, its life history can be characterized by low fecundity, delayed maturity, a long gestation period, and long lifespan. This species utilizes the shallow, sandy-bottomed habitat along the Sonoran coast for nursing grounds, and these areas are known to have a high proportion of gravid females from late summer to late winter [31], overlapping spatially and temporally with shrimp trawling. *R. productus* exemplifies the complex life history traits that are found in many of the sensitive and/or commercially important by-catch species observed in this study, and demonstrates the need for incorporating life history information into by-catch management. Removal of these and other valuable species represents present and future economic loss to small-scale fishers in the region.

This economic loss can be partially quantified by the increasing number of valuable by-catch species being retained by trawler crews to illegally sell to local fishers. In a single night, we observed 237 kg of nine taxa retained by trawler crew members; using price data gathered by interviewing local fishers, this amounts to roughly MN $2600. While this example represents direct losses to local fishers via decreased potential catch, it does not fully represent future losses due to decreased ecosystem function from the physical damage of trawling on the seabed, decreased recruitment of commercial fish due to high juvenile capture and mortality rates, and the loss of reproductively mature and gravid females landed in the by-catch. Future studies are recommended to quantify these economic and ecosystem impacts of shrimp trawler by-catch mortality.

Results presented here build upon findings from previous studies in the Gulf of California [6], [7], [14], [17], [20] highlighting a strong ecological and economic rationale for by-catch management within the trawl shrimp fishery of the Gulf of California. The specific need for by-catch management in the Bahía de Kino region is based on several factors:

1. By-catch reduction is imperative to restore the health of commercial fish populations. A by-catch management plan in the Bahía de Kino area is particularly indicated because the areas being trawled are in close proximity to Estero La Cruz; bays adjacent to estuaries are known to be nursery grounds for juvenile fish [32], and recently the importance of shallow coastal zones to commercial shark populations in Gulf of California has been described by Salomón-Aquilar et al. [33]. While future studies are needed to quantify the abundance of juveniles in the by-catch, our observations combined with literary references support the argument for an active by-catch management program in the region.
2. By-catch reduction is necessary to improve the economic health of fishing communities and to support community-based fisheries management initiatives gaining momentum in the Bahía de Kino region since 2000. Best practices in small-scale fisheries can only go so far with current levels of by-catch volume.
3. Effective by-catch management is critical to maintain the international political viability of the economically important export-based shrimp fishery.

Recent global initiatives prioritize shrimp fishery by-catch reduction and create the framework and incentive for the development of by-catch management plans [1], [2], [16]. Mexican fisheries managers helped craft the report of the FAO Expert Consultation on International Guidelines for Bycatch Management and Reduction of Discards calling for the adoption and implementation of by-catch management plans [16]. By-catch management plans in regional shrimp fisheries should consider:

1. Consistent on-board monitoring to quantify by-catch and increase compliance with existing **r**egulations.
2. Continued reduction of overcapitalized fleets.
3. Development and incentivizing the use of by-catch reduction device (BRD) technologies.
4. Establishment of no-trawl zones to support small-scale fishers and to allow for habitat and fish population recovery.
5. Evaluation of the application of catch shares or quotas for by-catch, preferably coupled with incentives for compliance.
6. Evaluation of the efficacy of temporal restrictions limiting trawling to 1–5am.

By-catch management is necessary to maintain the export-based shrimp fishery in a manner that does not cause undue burden to the human and ecological communities in the region. The high quality of wild-caught shrimp, the proximity to U.S. markets, lack of import duties on exports to the U.S., and certification under Section 609, make the Mexican shrimp commodity relatively profitable. The successful development and implementation of by-catch management plans in Sonora, as everywhere, will require the participation and investment at the industry and government level. Import/export companies and NGOs play an important role in education and marketing toward consumer support for more environmentally sustainably harvested shrimp products.

## Supporting Information

Table S1 **Appendix of by-catch species from sampled shrimp trawls in the Bahía de Kino region, 2003–2009.** Systematic list of by-catch species documented throughout the study period (2003–2009), along with year(s) of occurrence in the by-catch for each species.
(XLS)
